# Highly Efficient Blue Thermally Activated Delayed Fluorescence Emitters Based on Multi-Donor Modified Oxygen-Bridged Boron Acceptor

**DOI:** 10.3390/molecules27134048

**Published:** 2022-06-23

**Authors:** Xin-Yue Meng, Zi-Qi Feng, You-Jun Yu, Liang-Sheng Liao, Zuo-Quan Jiang

**Affiliations:** Institute of Functional Nano & Soft Materials, Jiangsu Key Laboratory for Carbon-Based Functional Materials & Devices, Joint International Research Laboratory of Carbon-Based Functional Materials and Devices, Soochow University, Suzhou 215123, China; 20204214056@stu.suda.edu.cn (X.-Y.M.); 20204214135@stu.suda.edu.cn (Z.-Q.F.); yi-yu@outlook.com (Y.-J.Y.); lsliao@suda.edu.cn (L.-S.L.)

**Keywords:** multi-donor, boron, TADF, high efficiency, OLEDs

## Abstract

The employment of thermally activated delayed fluorescence (TADF) emitters is one of the most promising ways to realize the external quantum efficiency (EQE) of over 25% for organic light-emitting diodes (OLEDs). In addition, the TADF emitter based on oxygen-bridged boron (BO) fragment can maintain blue emission with high color purity. Herein, we constructed two blue TADF emitters, 3TBO and 5TBO, for OLEDs application. Both emitters consist of three donors linked at the oxygen-bridged boron acceptor. OLED devices based on 3TBO and 5TBO exhibited both high excellent device efficiency and high color purity with a maximum EQE; full-width at half-maximum (FWHM); and CIE coordinates of 17.3%, 47 nm, (0.120, 0.294), and 26.2%, 57 nm, (0.125, 0.275), respectively.

## 1. Introduction

Recently, thermally activated delayed fluorescence (TADF) emitters have attracted enormous attention in optoelectronic applications [1], especially for organic light-emitting diodes (OLEDs) [2,3,4,5]. For TADF, the thermally activated triplet excitons can be up-converted to singlet excited excitons by a spin-flip process named reverse intersystem crossing (RISC). Then, the converted triplet excitons generate delayed fluorescence via radiative transition [6]. Through this process, TADF emitters are able to achieve near 100% internal quantum efficiency (IQE), which is one of the most promising ways to realize the high external quantum efficiency (EQE) in OLEDs. In 2012, Adachi et al. confirmed a small energy gap (Δ*E*_ST_) between S_1_ and T_1_ energy levels is essential for efficient RISC process [7]. To meet the requirement of small Δ*E*_ST_, TADF molecules based on twisted electron donor–acceptor (D-A) geometries are proposed because a large D-A-twisted angle promises a small overlap integral between highest occupied molecular orbital (HOMO) and lowest unoccupied molecular orbital (LUMO) [8,9].

To date, numerous D/A systems have been investigated [10,11,12,13,14]. As the donors are mainly aryl amines [15,16], the innovations of the building blocks are mostly concentrated on acceptors [14]. In 2015, a boron/oxygen-doped polycyclic aromatic hydrocarbon, BO (2a), was reported by Hatakeyama et al. [17]. The oxygen bridge in BO molecule skeleton allows it to maintain a wide band gap while maintaining rigidity. The multi-resonance effect of BO makes it have a small Δ*E*_ST_ (0.15 eV). When an electron-donating group was appended on it to construct a D-A type TADF, BO serving as an acceptor would maintain a weak electron-withdrawing ability and a rigid molecular configuration due to the electron-donating effect of its oxygen atom. Therefore, BO-based D-A-type molecules still maintain blue light emission with high color purity, which is usually measured by the full-width at half-maximum (FWHM). For example, in 2019, Kwon et al. reported the D-A derivatives TDBA-Ac and TDBA-DI [18]. Due to the rigid D-A structure, both TDBA-Ac and TDBA-DI had ideal photoluminescence quantum yield (PLQY) (93%/99%). In low-polarity host PPBI, TDBA-Ac achieved deep blue light (λ_EL_ = 448 nm, FWHM = 79 nm, EQE = 21.50%) and TDBA-DI achieved sky blue light luminescence (FWHM = 56 nm, EQE = 32.23%). Besides the modification at the *para* position of boron, its *meta* position had also been explored. In 2022, Yang et al. reported BO derivatives, TDBA-Cz and DBA-Cz, using 3,6-di-*tert*-butylcarbazole as the donor [19]. The doped devices based on TDBA-Cz and DBA-Cz achieved high efficiency and high color purity device performance (λ_EL_ = 466/467 nm, FWHM = 47/50 nm, EQE = 31.1%/30.3%), respectively. In order to further develop TADF emitters based on BO, it is feasible to introduce multiple donors. The construction of multi-donor has been applied in benzonitrile and 2,4,6-triphenyl-1,3,5-triazine-based TADF systems, such as the Cz-BN series [7,20] and Cz-TRZ series [21]. These previous studies prompt us to apply multi-donor construction in the BO TADF system.

In this work, we designed and synthesized two tri-donor TADF emitters, namely 3TBO and 5TBO, based on BO acceptors. They have a similar skeleton consisting of three 3,6-di-*tert*-butyl-9*H*-carbazole (tCz) units served as peripheral groups. As compared with 3TBO, two additional *tert*-butyl groups are attached to BO acceptor in 5TBO. Both emitters exhibited high efficiency and narrow emission, the FWHMs of electroluminescence (EL) were not only broadened but also shaped slightly as the doping concentration increased to 40 wt% by combining the three donor moieties. Compared with the 3TBO-based device, the device based on more coated 5TBO has better luminance performance and the maximal EQE increases from 17.2% to 26.2%.

## 2. Results

### 2.1. Molecular Synthesis

**Scheme 1:**
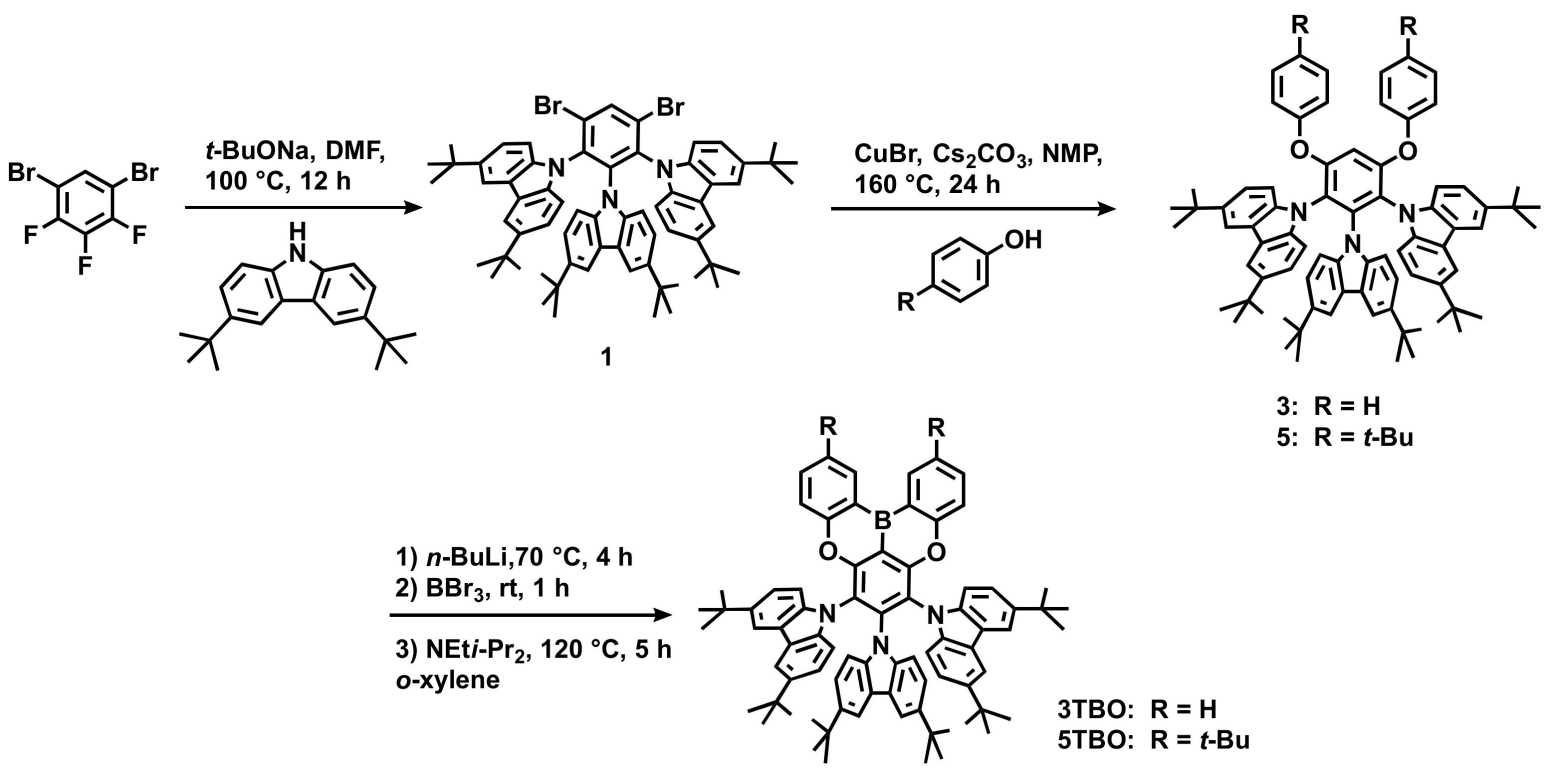
Synthetic routes for 3TBO and 5TBO.

The synthetic routes for 3TBO and 5TBO are depicted in Scheme 1. The detailed process is shown in Supporting Information. The starting material 1 was synthesized according to S_N_2Ar between tCz and 1,5-dibromo-2,3,4-trifluorobenzene in good yields. Then phenol or 4-(*tert*-butyl)phenol were subjected to Ullmann coupling to give the precursor 3 and 5, respectively. The target materials 3TBO and 5TBO were synthesized in one-pot borylation and confirmed by ^1^H NMR and ^13^C NMR spectroscopy, as well as mass spectrometry. 3TBO and 5TBO show good solubility in common organic solvents with the introduction of *tert*-butyl solubilizing group.

### 2.2. Thermal and Electrochemical Properties

The thermal properties of the emitters were evaluated by thermogravimetric analysis (TGA) and differential scanning calorimeter (DSC). The thermal decomposition temperature (*T*_d_) values with 5 wt% loss are 433 and 431 °C for 3TBO and 5TBO, respectively (see Supplementary Materials Figure S1). The excellent thermal stability makes them suitable candidates for the vacuum deposition process. The glass-transition temperature (*T*_g_) values of 3TBO and 5TBO are 216 and 228 °C, respectively.

Further, the HOMO energy levels of these materials were calculated from their oxidation potential. The bandgap values (*E_g_*) were estimated from the absorption onset. The LUMO values were calculated from the HOMO values and bandgap values. The *E_g_*s were 2.78 and 2.79 eV for 3TBO and 5TBO, respectively, indicating the *tert*-butyl has little impact on *E_g_*. Both 3TBO and 5TBO show the same oxidation peak for all five cycles (see Supplementary Materials Figure S2), which suggests that the coated structure ensures electrochemical stability of the two emitters [22].

### 2.3. Photophysical Properties

**Figure 1:**
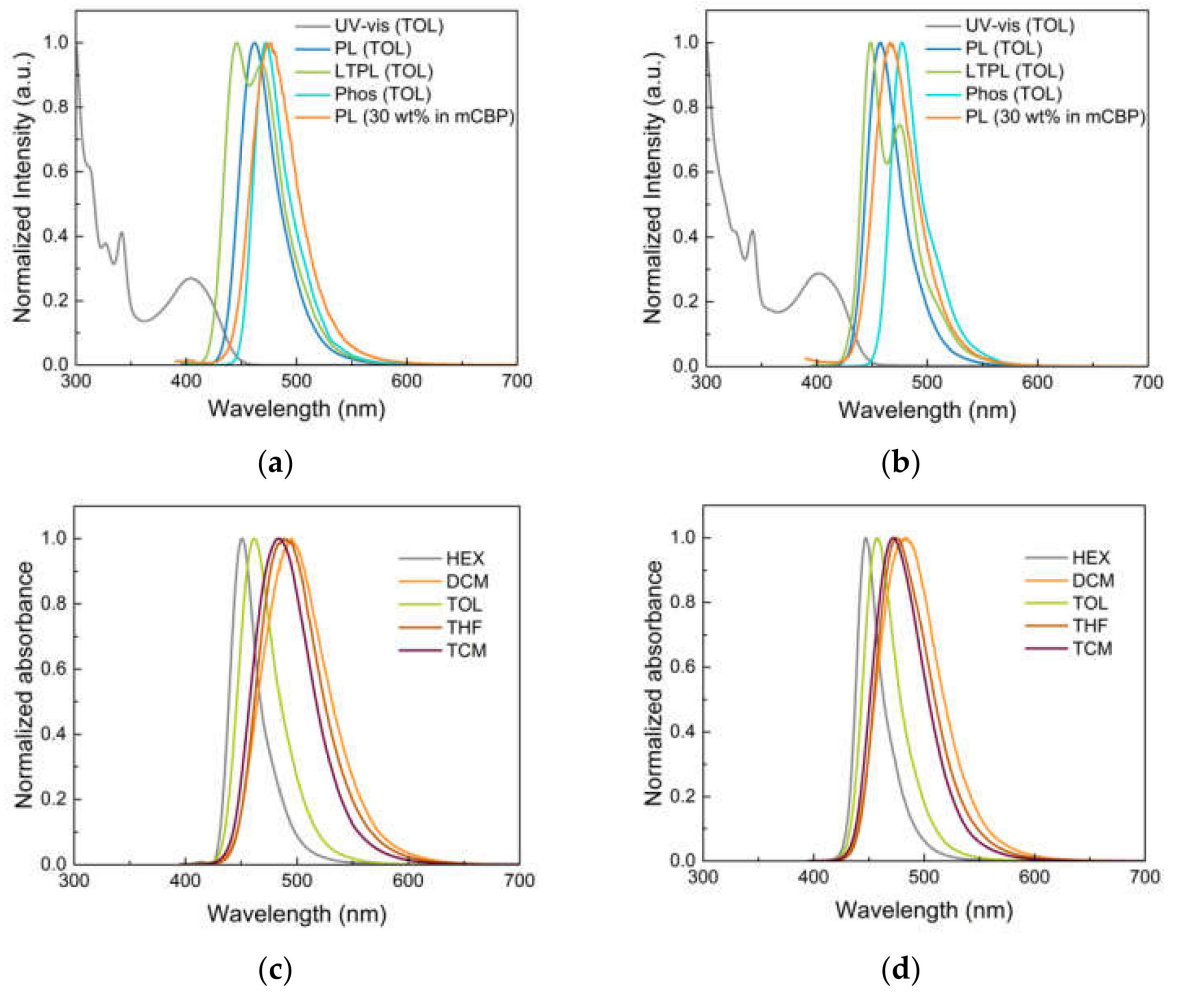

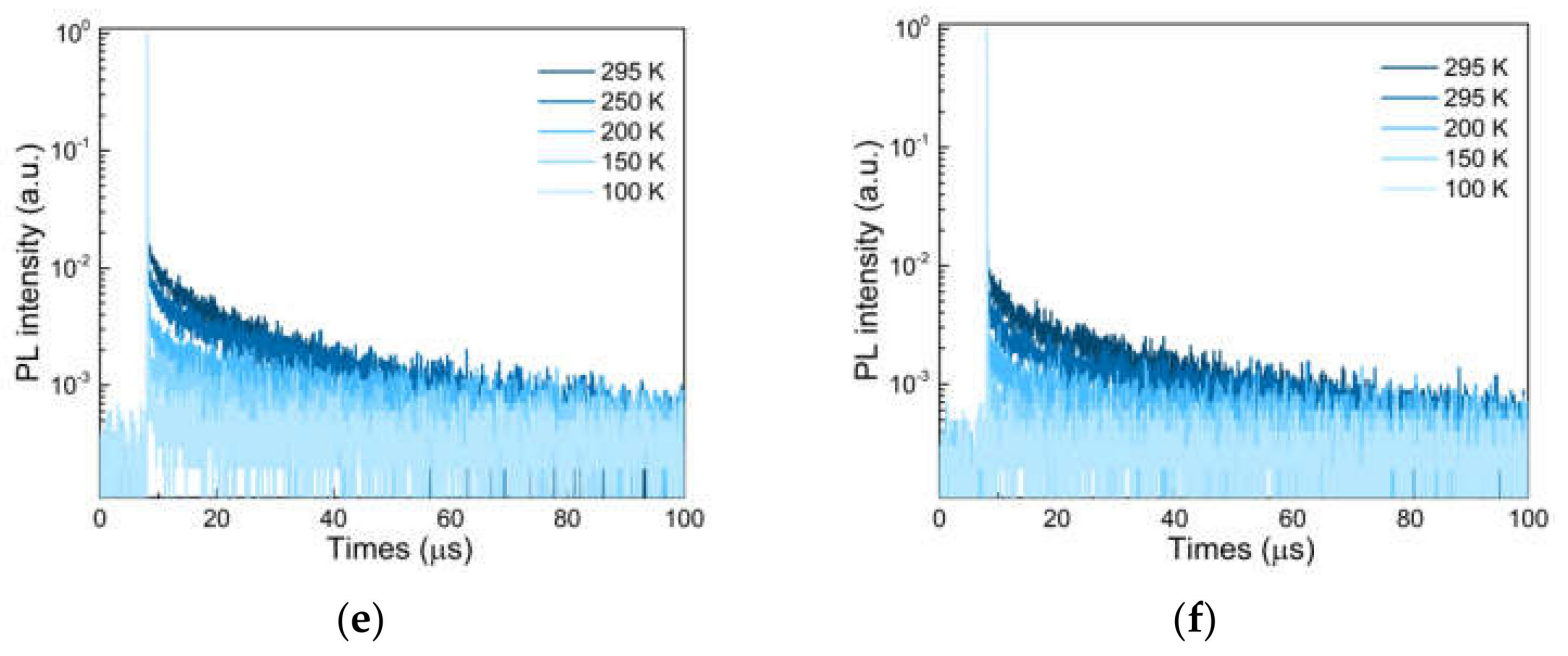
(**a**,**b**) Absorption spectrum, fluorescent (298 K), and phosphorescent spectra (78 K) in toluene (TOL) for 3TBO and 5TBO, respectively; (**c**,**d**) photophysical properties of 3TBO and 5TBO in various solutions with different polarities [hexane (HEX), Tol, THF, trichloromethane (TCM), dichloromethane (DCM)]; and (**e**,**f**) time-dependent transient PL decay characteristics of 3TBO and 5TBO in 30 wt% mCBP film from 100 to 295 K.

The solution- and film-state photophysical properties were tested by ultraviolet–visible (UV–vis) absorption and photoluminescence (PL), time-dependent transient PL decay. The photophysical properties of 3TBO and 5TBO in the solution and film states are shown in Figure 1 and summarized in Table 1 and Table 2.

With the introduction of tert-butyl group with electron-donating ability, the acceptor strength is slightly weakened [23]. Consequently, the maximum PL emission wavelength of 5TBO slightly blue shifts from 462 to 457 nm compared with 3TBO (Table 1). The steady-state spectral features of the investigated compounds are in Table 2. As can be seen from this table, the stokes shift (ν_st_) difference (812.5 cm^−1^) between LTPL and RTPL of 5TBO is 1.7 times smaller than that of 3TBO (1413.1 cm^−1^), indicating that the conformation relaxation of 5TBO from the ground state to excited state is smaller than that relaxation in 3TBO.

In Figure 1c,d, both emission wavelengths of 3TBO and 5TBO shift from blue to a green region with increasing solvent polarity, indicating their charge transfer (CT) characteristics in the lowest singlet excited state. This is consistent with the calculation results of the hole and electron distribution that will be discussed below. In particular, with increasing solvent polarity from HEX to DCM, 3TBO showed larger redshifts of 44 nm compared to the shifts of 36 nm for 5TBO, indicating that 3TBO has a stronger CT character than 5TBO. This can be the reason that 5TBO showed a bluer emission, although the introduction of *tert*-butyls did not change the energy gap apparently. The FWHM values of 3TBO and 5TBO were estimated to be 39 and 44 nm, respectively, which are the narrowest CT-TADF emitters.

The S_1_ and T_1_ energy levels of the emitters were determined from the peak of the fluorescence and phosphorescence spectra measured at 77 K in toluene (Figure 1a,b). Accordingly, the Δ*E*_ST_ values were established to be 0.15 and 0.16 eV for 3TBO and 5TBO, respectively. Such Δ*E*_ST_ values are sufficiently small to harvest triplet excitons through the RISC process, and as a result, the emitters are expected to exhibit TADF characteristics [24].

The temperature-dependent decay curves of the emitters were recorded for the 30 wt% doped 3,3′-di(9H-carbazol-9-yl)-1,1′-biphenyl (mCBP) films and shown in Figure 1e,f. The delayed portion increased with the increasing temperature for both materials, which indicates the existence of TADF properties [25,26]. Both materials showed bi-exponential decay corresponding to prompt and delayed emission. The detailed data were shown in Table 3 and Table S1. Due to the low Δ*E*_ST_ of the 3TBO, it undergoes a facile RISC process (9.03 × 10^4^ s^−1^). Thanks to the smaller conformation relaxation of 5TBO, the k_nr_^S^ (2.5 × 10^6^ s^−1^) is an order of magnitude smaller than the k_nr_^S^ of 3TBO (1.2 × 10^7^ s^−1^), which ensures the higher PLQY of 5TBO (96.7%) (Table 1). Hence, it is expected that 5TBO would show better TADF performance in its OLED device.

### 2.4. Theoretical Investigation

**Figure 2:**
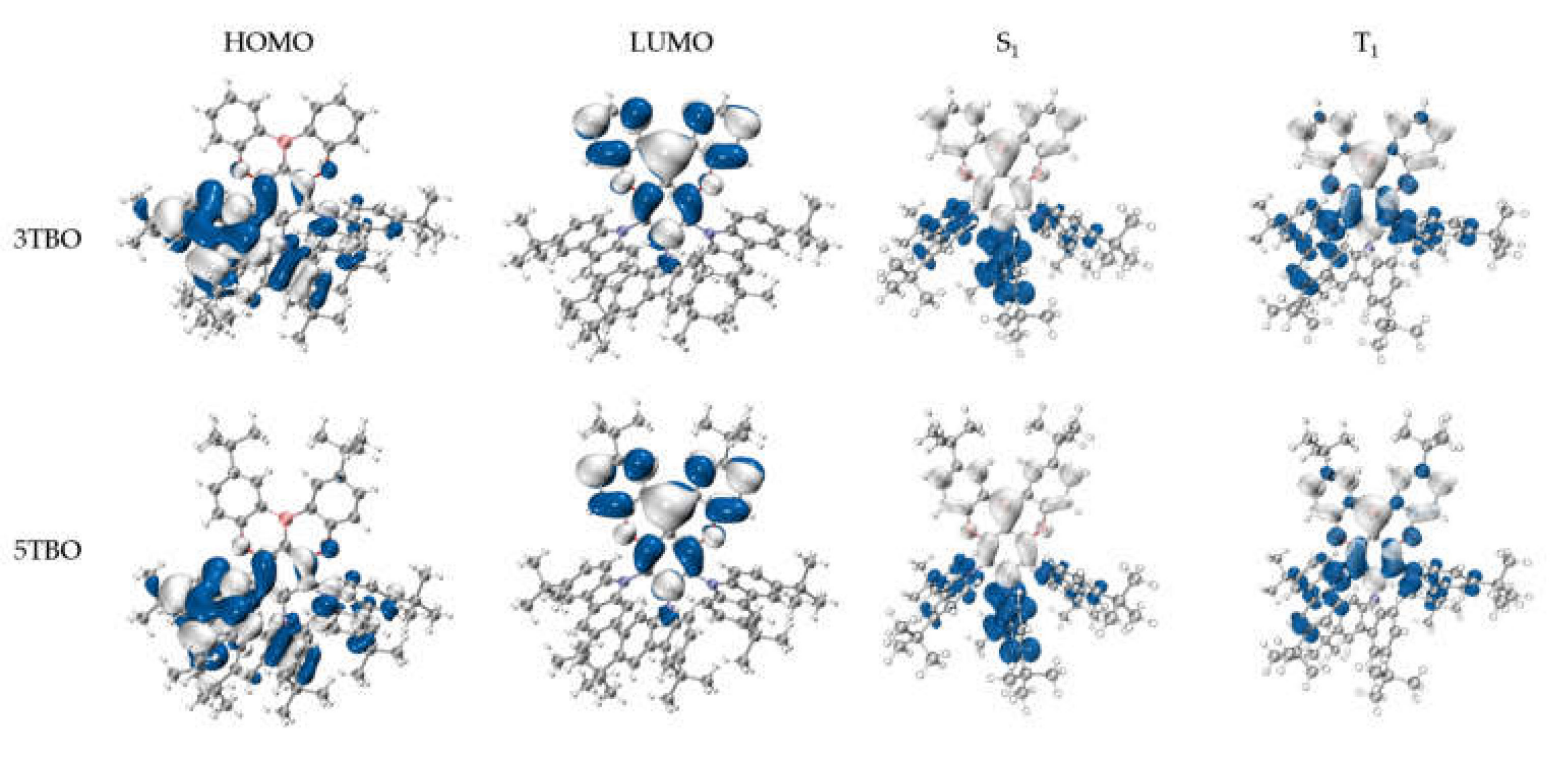
The distribution of the HOMOs and LUMOs and the analysis for the distribution of the hole (blue) and electron (white) for S_1_ and T_1_.

For 3TBO and 5TBO, Density Functional Theory (DFT) calculation was performed to analyze the ground state at B3LYP/def2-SVP level [27]. In addition, time-dependent DFT (TDDFT) was performed to analyze the electronic properties at PBE0/def2-SVP level [28] for the excited state in Gaussian 16. In Figure 2, the HOMO was unevenly located on the tCz donor with a small contribution to the acceptor segment. Both the molecules had similar HOMO levels of −5.23 and −5.19 eV, respectively. Also, the LUMO level of 3TBO (−2.17 eV) and 5TBO (−2.10 eV) were nearby. As shown in Figure 2, The LUMO was localized on the entire acceptor core. In addition, there was an overlapping area on the central benzene ring to ensure a high PLQY. The well-separated HOMO and LUMO orbitals would give a small Δ*E*_ST_. The calculated Δ*E*_ST_ was 0.19 and 0.22 eV for 3TBO and 5TBO, respectively. The small Δ*E*_ST_ of these materials could ensure good TADF performances. All the trends of calculated *E*_g_ and Δ*E*_ST_ were well marched with the experience. In Table 4, the oscillator strengths (*f*) of these materials were also calculated to be 0.0183 and 0.0216 for 3TBO and 5TBO, respectively. The difference in their oscillator strength may stem from their different electronic nature, which is tuned by the *tert*-butyl groups. In addition, the higher *f* of 5TBO promised a higher PLQY. The electron-hole analysis is also shown in Figure 2. The separated electron-hole isosurface demonstrated that both emitters are CT state in S_1_. Both molecules had similar electron-hole separation, which were not significantly affected by the *tert*-butyl units.

### 2.5. Device Performance

**Figure 3:**
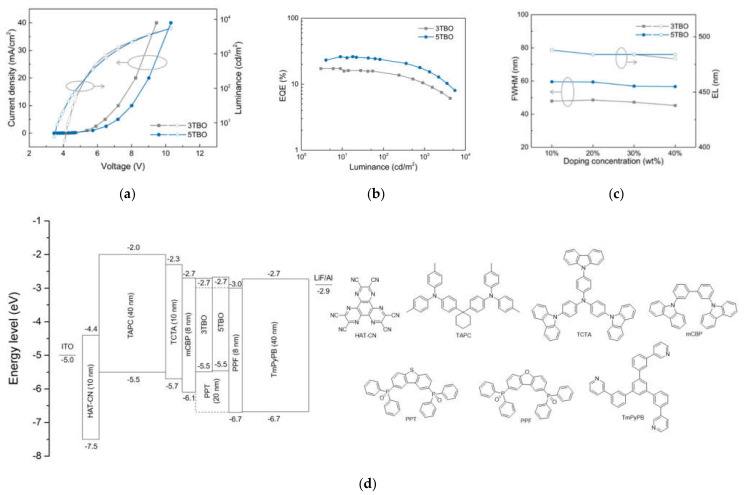
(**a**) Current density-voltage-luminance (*J*-*V*-*L*) characteristics; (**b**) external quantum efficiency versus current density (EQE-*J*) plots of 3TBO- and 5TBO-based OLEDs; (**c**) the FWHM and EL of the doped films with different dopant concentrations; (**d**) device structures of OLEDs based on 3TBO and 5TBO, and molecular structures of organic functional materials.

Employing 3TBO and 5TBO as emitters, two multi-layered OLEDs were fabricated using the following device architecture (Figure 3d): [ITO (indium tin oxide)/1,4,5,8,9,11-hexaazatriphenylenehexacarbonitrile (HAT-CN) (10 nm)/1,1-bis[(di-4-tolylamino)phenyl]cyclohexane (TAPC) (40 nm)/4,4′,4″-tris-(carbazol-9-yl)-triphenylamine (TCTA) (10 nm)/mCBP (8 nm)/2,8-bis(diphenylphosphoryl)dibenzo[b,d]thiophene (PPT):emitters (x wt%) (20 nm)/2,8-bis(diphenylphosphoryl)dibenzo[b,d]furan (PPF) (8 nm)/1,3,5-tri[(3-pyridyl)-phen-3-yl] benzene (TmPyPB) (40 nm)/8-hydroxyquinolinolato-lithium (Liq) (2.5 nm)/Al (60 nm)] were fabricated (x = 10, 20, 30, 40), where HAT-CN is employed as the hole injection layer, TAPC is used as hole-transporting layer, and TCTA and mCBP bilayers with relatively high triplet energy levels are embedded between TAPC and emitting layer. Besides electron- and exciton-blocking capability, herein they are also beneficial for the carrier injection to the emitting layer due to the intermediate HOMO levels between the TAPC and PPT matrix, so that cascade HOMO levels could effectively reduce the driving voltage, as well as the energy loss during the carrier injection and transporting process owing to the large energy level gap. Following the emitting layer, 8-nm-thickness PPF neat film is used to confine the generated excitons within the light-emitting layer, then, TmPyPB and Liq are served as electron-transporting and electron-injection layers, respectively. The energy level diagram of the structure of the materials used for the fabrication of the devices is shown in Figure S3. The electroluminescence performances of these materials are shown in Figure 3 and Figures S3–S4, and the data are presented in Table 5.

Figure 3a,b shows the current density-voltage-luminance (*J*-*V*-*L*) and EQE versus current density (*J*) plots. The 5TBO-based device exhibited turn-on voltages (V_on_) of 3.46 V, which is lower than that of 3TBO (4.08 V). This result indicated a more balanced charge collection in the emissive layer of 5TBO-based devices. The 5TBO-based device with a 30 wt% doping concentration achieved the maximum EQE of 26.2%. As comparison, the 3TBO-based device exhibited a relatively low efficiency with a maximum EQE of only 17.3%. This is primarily attributed to the relatively higher PLQY of 5TBO, which was discussed above. In addition, this trend is consistent with the earlier research by Lee [29]. Compared with the TB-tCz-device reported by Choi in 2021 [30], the device efficiency of these tri-donor BO emitters was significantly improved. As shown in Figure 3c, all the devices exhibited blue EL emission. The EL spectra of both emitters in 30 wt% doped devices were redshifted and broadened compared with solvent state, which may be ascribed to the polar nature of different medium. Thanks to the coated skeleton of 5TBO, as the doping concentration increased, the emission maxima only shifted slightly from 488 to 484 nm and the FWHM narrowed from 60 to 57 nm (Figure 3c). The EL and FWHM based on these two emitters are not sensitive to concentration changes.

## 3. Conclusions

In summary, we designed and synthesized two blue TADF emitters, namely 3TBO and 5TBO, based on BO acceptors. They both possess twisted tri-carbazole groups, forming spatial steric effect. As a result, they FWHMs in EL spectra are steady while the doping concentration increased. When applied in OLEDs, both devices exhibit narrow and blue emissions. Specifically, 5TBO, with two additional *tert*-butyl groups, achieved a high PLQY of 97% and good device EQE of 26.2%, which are superior to 3TBO. Finally, 5TBO-based OLED exhibited not only a smaller CIE_y_ (0.275) but also a higher EQE (26.2%).

## Figures and Tables

**Table 1:** Summary of the physical properties of 3TBO and 5TBO.

Molecules	PL/FWHM (TOL, nm)	PL/FWHM (30 wt% mCBP, nm)	*E*_S_ (eV)	*E*_T_ (eV)	Δ*E*_ST_ (eV)	PLQY (30 wt% mCBP, %)
3TBO	462 ^1^/39	476 ^1^/47	2.78	2.63	0.15	78.4
5TBO	457 ^1^/44	466 ^1^/43	2.76	2.60	0.16	96.7

^1^ At room temperature.

**Table 2:** Steady-state spectral features of 3TBO and 5TBO in toluene.

	Molecules	λ(nm)	ν (cm^−1^)	ν_st_ (cm^−1^)
UV-vis	3TBO	405	24,691.4	
	5TBO	402	24,875.6	
PL (298 K)	3TBO	462	21,645.0	3046.3
	5TBO	457	21,881.8	2993.8
LTPL (78 K)	3TBO	446	22,421.5	2269.8
	5TBO	449	22,271.7	2603.9

**Table 3:** Rates of the photophysical processes of 3TBO and 5TBO ^1^.

Molecules	*k*_p_ (10^7^ s^−1^)	*k*_d_ (10^4^ s^−1^)	Φ_PF_ (%)	Φ_DE_ (%)	Φ_ISC_ (%)	*k*_r_^S^ (10^7^ s^−1^)	*k*_nr_^S^ (10^7^ s^−1^)	*k*_ISC_ (10^7^ s^−1^)	*k*_RISC_ (10^4^ s^−1^)
3TBO	7.16	7.03	61.1	17.3	22.1	4.37	1.20	1.58	9.03
5TBO	8.51	5.26	86.2	10.5	10.8	7.34	0.25	0.92	5.89

^1^ Φ_DF_, Φ_PF_, *k*_p_, and *k*_d_—PL efficiencies and decay rates for the prompt and delayed emissions; *k*_ISC_, *k*_RISC_, and *k*_r_—rates of intersystem crossing, reverse intersystem crossing, and radiative deactivation, respectively; *k*_nr_^s^—rates of nonradiative deactivation excluding (r)ISC of the singlet states.

**Table 4:** DFT and TD-DFT calculation results.

Molecules	HOMO (eV)	LUMO (eV)	*E*_g_ (eV)	*S*_1_ (eV)	*T*_1_ (eV)	Δ*E*_ST_ (eV)	*f*
3TBO	−5.23	−2.17	3.06	2.73	2.53	0.19	0.0183
5TBO	−5.19	−2.10	3.09	2.76	2.54	0.22	0.0216

**Table 5:** Device performance of 3TBO- and 5TBO-based OLEDs ^1^.

Emitters	V_on_ (V)	Max. Luminance (cd/m^2^)	CE Max/@1000 cd/m^2^ (cd/A)	EQE Max/@1000 cd/m^2^ (%)	EL (nm)	FWHM (nm)	CIE
3TBO	4.1	4249	30.0/17.7	17.3/10.3	484	47	(0.120, 0.294)
5TBO	3.5	5544	45.0/28.5	26.2/16.7	484	57	(0.125, 0.275)

^1^ With 30 wt% dopant concentration.

## Data Availability

Data is contained within the article or Supplementary Materials.

## Supplementary Materials

The following supporting information can be downloaded at: https://www.mdpi.com/article/10.3390/molecules27134048/s1. Synthesis and characterization; Figure S1: (a) TGA thermograms and (b) DSC analysis of 3TBO and 5TBO at a heating rate of 10 °C min^−1^ under N_2_; Figure S2: Multiple scan cyclic voltammograms for the oxidation of (a) 3TBO and (b) 5TBO emitters in dichloromethane solutions at RT, respectively; Figure S3: (a) *J-V-L* curves, (b) CE-*L*-PE curves, (c) EL spectra, and (d) EQE-*L* curves of 3TBO-doped devices at various dopant concentrations; Figure S4: (a) *J-V-L* curves, (b) CE-*L*-PE curves, (c) EL spectra, and (d) EQE-*L* curves of 5TBO-doped devices at various dopant concentration; Figure S5: Time-dependent transient PL decay curves of 3TBO and 5TBO in 30 wt% mCBP film at 295 K; Figure S6: ^1^H NMR spectrum of 3TBO in CDCl_3_; Figure S7: ^13^C NMR spectrum of 3TBO in CDCl_3_; Figure S8: ^1^H NMR spectrum of 5TBO in CDCl_3_; Figure S9: ^13^C NMR spectrum of 5TBO in CDCl_3_; Table S1: Fitting data for transient PL decay curves of 3TBO and 5TBO; DFT calculation.

## Sample Availability

Samples of the compounds are not available from the authors.
