# Supplementary material for: Highly Efficient Blue Thermally Activated Delayed Fluorescence Emitters Based on Multi-Donor Modified Oxygen-Bridged Boron Acceptor
Source: Molecules. 2022 Jun 23;27(13):4048. doi: 10.3390/molecules27134048 (PMC9268263; doi:10.3390/molecules27134048)
Supplement: Supplementary file 1 [file molecules-27-04048-s001.zip › molecules-1782049-supplementary.pdf]

molecules

Supplementary Materials

Highly Efficient Blue Thermally Activated Delayed Fluorescence Emitters Based on Multi-Donor Modified Oxygen-Bridged Boron Acceptor

Xin-Yue Meng †, Zi-Qi Feng †, You-Jun Yu, Liang-Sheng Liao and Zuo-Quan Jiang *

Institute of Functional Nano & Soft Materials, Jiangsu Key Laboratory for Carbon-Based Functional Materials & Devices, Joint International Research Laboratory of Carbon-Based Functional Materials and Devices, Soochow University, Suzhou 215123, China

† These authors contributed equally to this work.
* zqjiang@suda.edu.cn

Contents

1. General information
2. Synthesis and characterization
3. Thermal and electrochemical properties
4. Device fabrication and characterization
5. Transient photoluminescence
6. DFT calculation
7. NMR spectroscopy
8. Reference

1. General information

All of the reagents and solvents were obtained from commercial sources and directly used without any further purification. ^{1}H NMR and ^{13}C NMR were measured in $CDCl_3$ solutions by an Agilent BRUKER AVANCE NEO 400MHz NMR spectrometer at 298K with chemical shifts (δ, ppm) relative to tetramethyl silane (Me_4Si) for the ^{1}H NMR and ^{13}C NMR spectra. Mass spectroscopy was performed using a Thermo Fisher ISQ Single Quadrupole MS 7000. Ultraviolet-visible absorption spectra were measured by a Shimadzu UV-2600 spectrophotometer. Fluorescent and phosphorescent spectra were measured by a Hitachi F-4600 spectrophotometer. Absolute PL quantum yields (PLQY) were measured on a Quantaurus-QY measurement system (C11347-11, Hamamatsu Photonics) under nitrogen flow and all samples were excited at 360 nm. Electrochemical analysis was carried out by a CHI604D instrument in a conventional three-electrode configuration system: glassy carbon electrode as working electrode, Ag/AgCl electrode as reference electrode and Pt wire electrode as counter electrode. The oxidative scans were performed using 0.1 M Bu_4NPF_6 in dichloromethane (DCM) as the supporting electrolyte and a scan rate of 100 mV s^{-1} at room temperature. Reductive scans were performed using 0.1 M Bu_4NPF_6 in dimethylformamide (DMF) as the supporting electrolyte and a scan rate of 100 mV s^{-1} at room temperature. The ferrocenium/ferrocene couple (Fc^{+}/Fc) was used as the internal reference. Thermogravimetric analysis (TGA) was performed by a METTLER TOLEDO TGA1 under nitrogen atmosphere. The temperature was increased to 700 °C with a heating rate of 10 °C min^{-1}. Differential scanning calorimetry (DSC) measurements were performed by a METTLER TOLEDO DSC1 under nitrogen atmosphere. The temperature was increased and decreased with a heating or cooling rate of 10°C min^{-1}. The measurements were repeated 2 times and 2nd cycle was utilized to determine the glass transition temperature. The melting points of compounds are measured by BUCHI M565 melting point instrument. The transient PL decay characteristics of deoxygenated solution samples were recorded using a Quantaurus-Tau fluorescence lifetime measurement system (C11367-03, Hamamatsu Photonics).

2. Synthesis and characterization

9,9',9''-((1*r*,2*r*,3*s*)-4,6-dibromobenzene-1,2,3-triyl)tris(3,6-di-*tert*-butyl-9*H*-carbazole) (1):

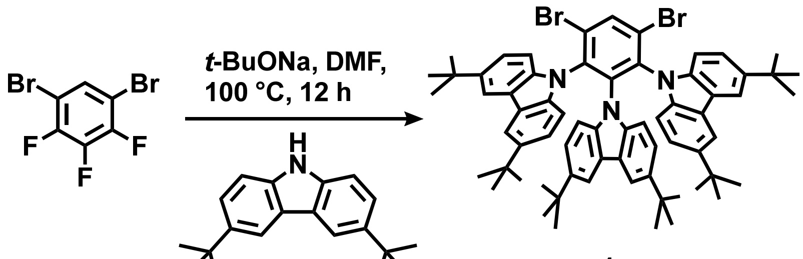

1

The 3,6-di-*tert*-butyl-9*H*-carbazole (3.88 g, 13.89 mmol) and *t*BuONa (1.33 g, 31.89 mmol) were dissolved in 126 mL of DMF. The mixture was stirred at room temperature for 30 min. After 1.22 g (4.21 mmol) of 1,5-dibromo-2,3,4-trifluorobenzene was added, the mixture was heated with stirring to 100 ºC for 12 h. The reaction mixture was cooled to room temperature and poured into a large amount of ice water. The aqueous reaction mixture was extracted with dichloromethane and the combined organic extracts were dried (magnesium sulphate). After filtering to remove the desiccant, the crude product was purified by silica gel column chromatography (elution with petroleum-dichloromethane, 9: l) to obtain the **1** (4.16 g, 92.54%). ^{1}H NMR (400 MHz, Chloroform-*d*) δ 8.49 (s, 1H), 7.65 – 7.57 (m, 4H), 7.15 – 7.09 (m, 2H), 7.02 (dd, J = 8.7, 1.8 Hz, 4H), 6.91 (d, J = 8.6 Hz, 4H), 6.60 (d, J = 8.6 Hz, 2H), 6.51 (dd, J = 8.7, 1.9 Hz, 2H), 1.30 (s, 36H), 1.17 (s, 18H). MS (MALDI-TOF) m/z calculated for $C_{66}H_{73}Br_2N_3$: 1067.42, found: 1067.33 $[M]^{+}$.

9,9',9''-((1*r*,2*r*,3*s*)-4,6-diphenoxybenzene-1,2,3-triyl)tris(3,6-di-*tert*-butyl-9*H*-carbazole) (3):

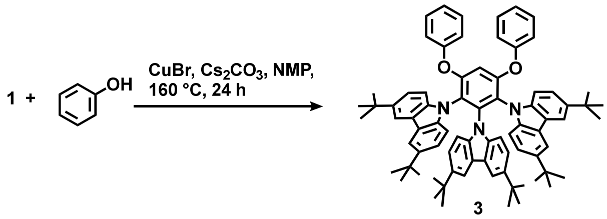

3

In a 100-mL two-necked, round-bottomed flask and fitted with a stirrer and a condenser tube, a mixture of **1** (4.16 g, 3.89 mmol), phenol (0.96 g, 10.13 mmol), cesium carbonate (2.79 g, 8.57 mmol), powdered copper(I) bromide (16.76 mg, 2.5 mol %) were dissovled in 23.24 mL of NMP under anhydrous and Ar condition. The reaction was placed in a preheated oil bath at 160 °C for 16 h. After cold to room temperature, the reaction mixture was filtered with suction, washed with dichloromethane and then removed the solvent *in vacuo*. The crude product was purified by silica gel column chromatography (elution with petroleum-dichloromethane, 9: 1) to give the **3** (3.06 g, 71.78%). ^{1}H NMR (400 MHz, Chloroform-*d*) δ 7.56 (s, 4H), 7.19 (d, J = 1.9 Hz, 2H), 7.09 – 6.99 (m, 13H), 6.84 (dd, J = 23.5, 8.0 Hz, 4H), 6.76 (d, J = 8.1 Hz, 4H), 6.57 (dd, J = 8.6, 1.9 Hz, 2H), 1.31 (s, 36H), 1.21 (s, 18H). MS (MALDI-TOF) m/z calculated for $C_{78}H_{83}N_3O_2$: 1093.65, found: 1093.83 $[M]^+$.

9,9’,9’’-((1*r*,2*r*,3*s*)-4,6-bis(4-(*tert*-butyl)phenoxy)benzene-1,2,3-triyl)tris(3,6-di-*tert*-butyl-9*H*-carbazole) (5):

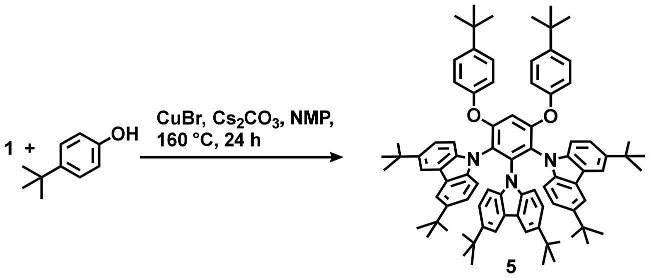

5

In a 100-mL two-necked, round-bottomed flask and fitted with a stirrer and a condenser tube, a mixture of **1** (3.00 g, 2.81 mmol), 4-(*tert*-butyl)phenol (1.10 g, 7.30 mmol), cesium carbonate (2.01 g, 6.18 mmol), powdered copper(I) bromide (12.09 mg, 2.5 mol %) were dissovled in 15.00 mL of NMP under anhydrous and Ar condition. The reaction was placed in a preheated oil bath at 160 °C for 16 h. After cold to room temperature, the reaction mixture was filtered with suction, washed with dichloromethane and then removed the solvent *in vacuo*. The crude product was purified by silica gel column chromatography (elution with petroleum-dichloromethane, 9: 1) to give the **5** (2.37 g, 69.93%). MS (MALDI-TOF) m/z calculated for $C_{86}H_{99}N_3O_2$: 1205.77, found: 1205.46 $[M]^+$.

9,9’,9’’-(5,9-dioxa-13b-boranaphtho [3,2,1-*de*]anthracene-6,7,8-triyl)tris(3,6-di-*tert*-butyl-9*H*-carbazole) (3TBO):
3TBO was synthesized according to literatures[17]. ^{1}H NMR (400 MHz, Chloroform-*d*) δ 8.77 (dd, J = 7.8, 1.7 Hz, 2H), 7.76 (dd, J = 1.8, 0.6 Hz, 4H), 7.59 (ddd, J = 8.6, 7.1, 1.7 Hz, 2H), 7.42 (ddd, J = 8.1, 7.1, 1.1 Hz, 2H), 7.24 (d, J = 1.9 Hz, 2H), 7.00 (d, J = 1.9 Hz, 1H), 6.97 (d, J = 1.9 Hz, 3H), 6.96 (d, J = 1.1 Hz, 1H), 6.91 (dd, J = 8.6, 0.6 Hz, 4H), 6.80 (d, J = 8.6 Hz, 2H), 6.56 (dd, J = 8.6, 2.0 Hz, 2H), 1.35 (s, 36H), 1.22 (s, 18H). ^{13}C NMR (101 MHz, Chloroform-*d*) δ 160.19, 152.90, 142.35, 142.27, 139.79, 138.85, 137.41, 134.42, 134.13, 124.13, 123.78, 123.64, 122.65, 121.95, 119.05, 117.85, 115.43, 114.63, 110.18, 110.10, 77.33, 77.01, 76.69, 34.50, 34.24, 31.95, 31.79. MS (MALDI-TOF) m/z calculated for $C_{78}H_{80}BN_3O_2$: 1101.63, found: 1101.54 $[M]^+$.

9,9’,9’’-(2,12-di-*tert*-butyl-5,9-dioxa-13b-boranaphtho [3,2,1-*de*]anthracene-6,7,8-triyl)tris(3,6-di-*tert*-butyl-9*H*-carbazole) (5TBO):

5TBO was synthesized according to literatures[17]. ^{1}H NMR (400 MHz, Chloroform-*d*)) δ 8.81 (d, *J* = 2.4 Hz, 2H), 7.77 – 7.72 (m, 4H), 7.64 (dd, *J* = 8.9, 2.4 Hz, 2H), 7.23 (d, *J* = 2.0 Hz, 2H), 7.01 – 6.87 (m, 10H), 6.79 (dd, *J* = 8.6, 2.1 Hz, 2H), 6.54 (dd, *J* = 8.6, 1.9 Hz, 2H), 1.48 (s, 18H), 1.35 (d, *J* = 0.9 Hz, 36H), 1.22 (d, *J* = 0.9 Hz, 18H). ^{13}C NMR (101 MHz, Chloroform-*d*) δ 145.87, 142.22, 142.15, 138.86, 137.44, 131.93, 130.04, 123.70, 122.59, 121.88, 118.50, 117.57, 115.37, 114.56, 110.22, 110.13, 34.63, 34.49, 34.23, 31.96, 31.79, 31.52. MS (MALDI-TOF) m/z calculated for $C_{86}H_{96}BN_3O_2$: 1213.76, found: 1213.92 $[M]^+$.

3. Thermal and electrochemical properties

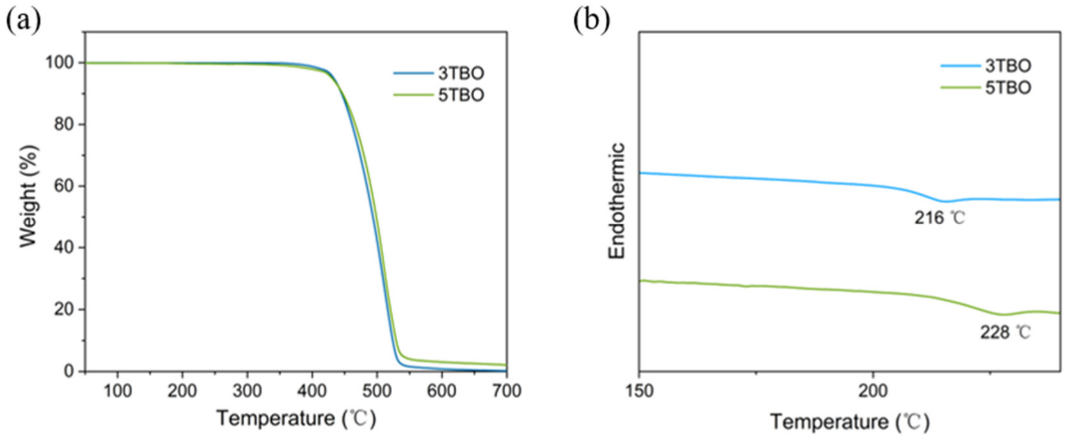

Figure S1. (a) TGA thermograms and (b) DSC analysis of 3TBO and 5TBO at a heating rate of 10 ^{o}C min^{-1} under N_2.

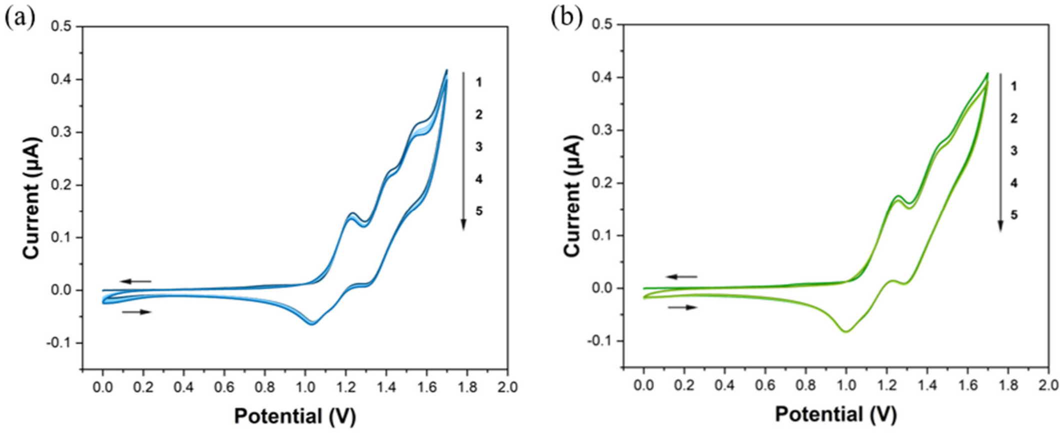

Figure S2. Multiple scan cyclic voltammograms for the oxidation of (a) 3TBO and (b) 5TBO emitters in dichloromethane solutions at RT, respectively.

4. Device fabrication and characterization

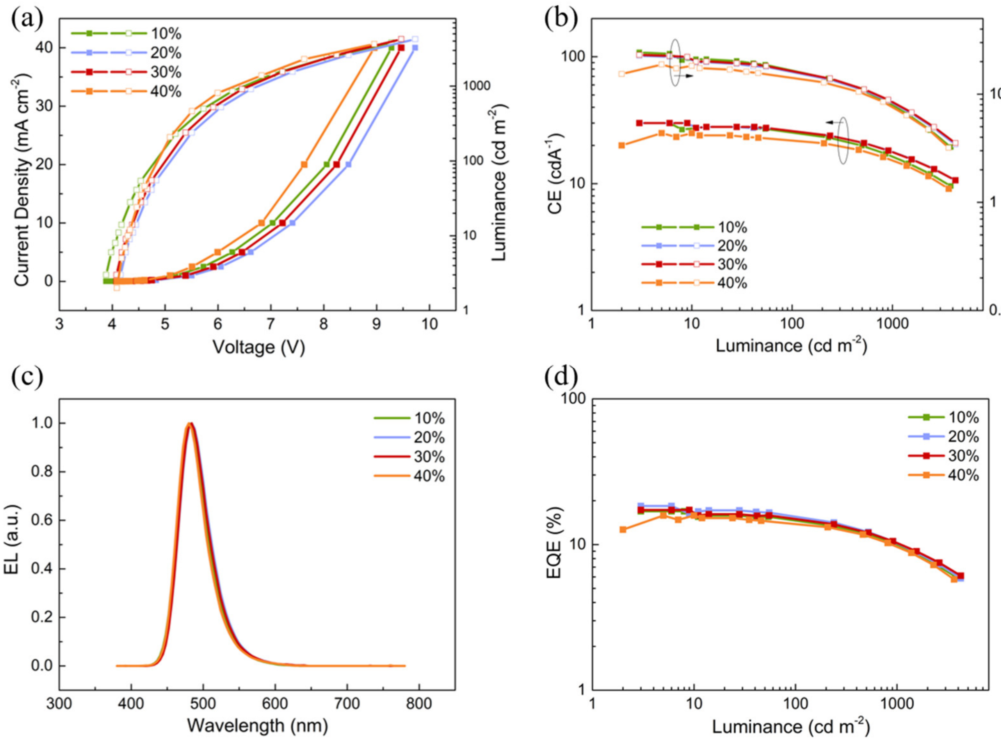

Figure S3. (a) *J*–*V*–*L* curves, (b) CE-*L*-PE curves, (c) EL spectra and (d) EQE–*L* curves of 3TBO-doped devices at various dopant concentration.

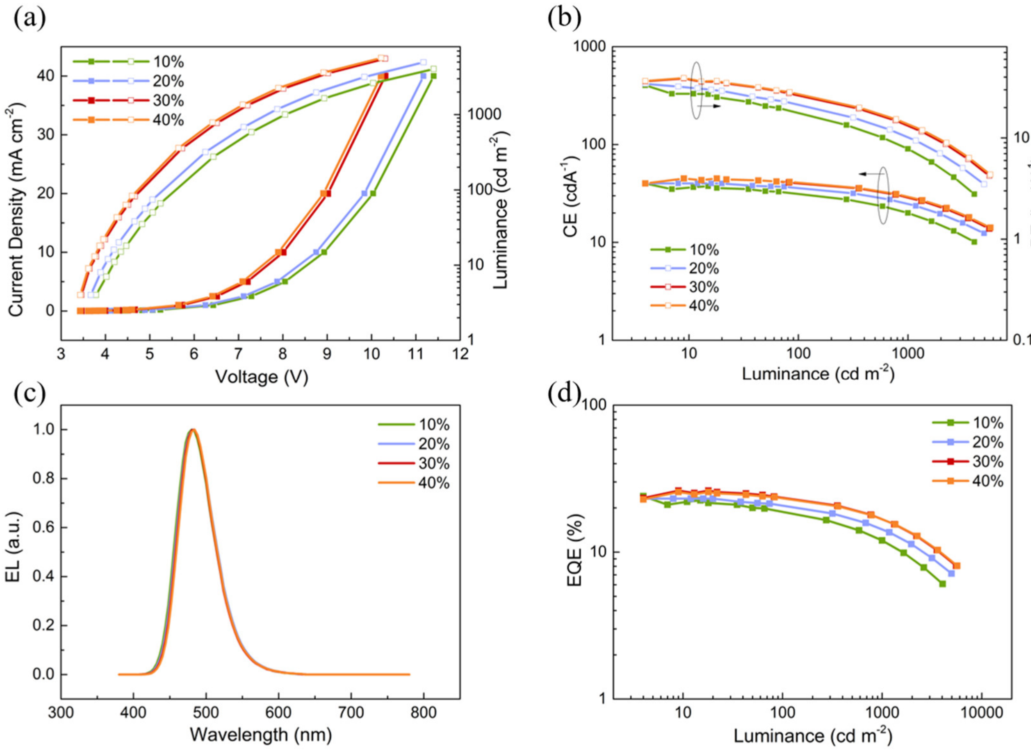

Figure S4. (a) *J*–*V*–*L* curves, (b) CE-*L*-PE curves, (c) EL spectra and (d) EQE–*L* curves of 5TBO-doped devices at various dopant concentration.

5. Transient photoluminescence

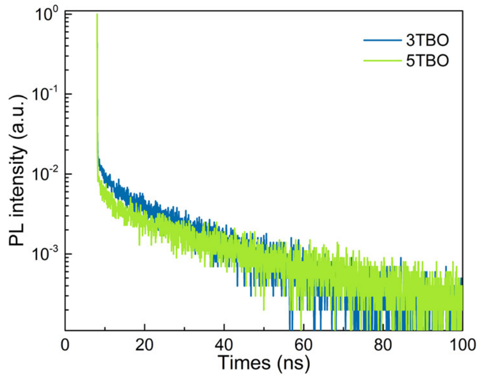

Figure S5. Time-dependent transient PL decay curves of 3TBO and 5TBO in 30 wt% mCBP film at 295 K.

Table S1. Fitting data for transient PL decay curves of 3TBO and 5TBO.

Dopant	τ_p/s	τ_d/s
3TBO	1.40E-08	1.42E-05
5TBO	1.18E-08	1.90E-05

6. DFT calculation

All density functional theory (DFT) calculation was carried out using Gaussian 16 C.01 software package[31]. The gas-phase ground state geometry optimizations were performed at B3LYP/def2-SVP level. Time-dependent density functional theory (TD-DFT) calculation was adopted at PBE0/def2-SVP level to optimize geometries of excited states. Based on the results of TD-DFT calculation, hole-electron analysis was performed using Multiwfn 3.8 software package[32] and VMD 1.9.3[33].

Cartesian coordinates of optimized 3TBO:

O 5.02050000 16.82850000 15.45670000
O 2.75910000 12.73190000 14.41820000
N 1.82220000 12.51320000 16.91060000
N 2.66120000 14.47930000 18.88750000
N 4.42590000 16.54670000 18.10100000
C 4.25910000 15.74960000 15.79500000
C 2.60190000 13.29610000 19.65090000
C 0.06260000 11.37330000 17.82460000
C 0.55450000 12.64720000 17.47580000
C 3.89010000 14.80420000 14.83260000
C 3.13750000 13.71850000 15.28000000
C 2.69640000 13.58280000 16.58710000
C 3.09140000 14.55310000 17.53690000
C 3.92030000 15.62550000 17.14430000
C 1.06940000 10.41210000 17.41790000
C 5.75720000 17.41350000 19.76030000
C 5.30950000 16.22450000 19.14490000

C 3.28900000 12.10350000 19.48100000
H 3.98700000 12.02430000 18.84070000
C 6.66340000 17.36070000 20.80930000
H 6.96010000 18.16700000 21.21430000
C 4.32490000 17.94930000 18.03100000
C -1.17520000 11.25560000 18.43380000
H -1.50470000 10.39620000 18.66970000
C 1.72590000 15.38890000 19.45800000
C 2.13690000 11.13970000 16.87050000
C -1.94210000 12.38330000 18.70310000
C 5.12900000 18.50890000 19.03460000
C 1.66530000 13.44580000 20.67390000
C 1.30970000 16.63140000 19.00400000
H 1.73310000 17.05460000 18.26620000
C -0.20730000 13.78590000 17.68080000
H 0.10560000 14.63940000 17.40460000
C 2.92290000 11.03060000 20.28040000
H 3.36920000 10.20120000 20.15720000
C 5.18440000 19.89400000 19.20380000
H 5.72510000 20.26980000 19.88820000
C 1.11320000 14.78010000 20.56670000
C 6.65050000 14.97400000 20.64830000
H 6.95680000 14.13270000 20.96610000
C 5.30570000 17.13120000 14.13360000
C 5.75050000 14.99090000 19.59930000
H 5.44440000 14.18380000 19.20260000
C 1.15660000 9.02450000 17.53320000
H 0.42750000 8.53340000 17.89340000
C -1.43350000 13.64470000 18.29750000
H -1.95740000 14.42110000 18.45620000
C 7.13900000 16.14570000 21.27300000
C 4.99660000 16.27950000 13.05430000
C 3.28810000 10.49960000 16.44250000
H 4.01240000 10.98790000 16.06780000
C 3.24960000 12.66450000 13.12020000
C 1.92570000 11.11140000 21.26050000
C 3.57750000 18.75640000 17.18440000
H 3.03590000 18.38110000 16.50020000
C 3.64840000 20.11620000 17.36890000
H 3.13660000 20.67650000 16.79710000
C 4.44230000 20.72130000 18.36590000

C 0.06270000 15.41740000 21.22060000
H -0.33670000 15.00620000 21.97830000
C 2.29960000 8.36100000 17.12560000
C 0.25180000 17.23200000 19.67020000
H -0.04720000 18.08150000 19.36720000
C 1.34410000 12.36010000 21.48740000
H 0.72600000 12.47060000 22.20040000
C -3.27690000 12.31420000 19.44480000
C 5.97220000 17.98800000 11.60330000
H 6.20190000 18.28540000 10.73100000
C 3.97340000 13.70690000 12.51460000
C -0.40900000 16.64460000 20.77970000
C 3.34830000 9.12410000 16.58050000
H 4.13360000 8.67390000 16.29330000
C 2.46380000 6.84030000 17.25940000
C 5.34830000 16.77340000 11.77460000
H 5.14620000 16.24860000 11.00880000
C 5.93520000 18.36270000 13.98250000
H 6.13460000 18.90200000 14.73880000
C 1.44240000 9.88870000 22.05510000
C 6.26420000 18.78330000 12.71320000
H 6.69540000 19.62140000 12.59150000
C 4.44550000 22.24810000 18.51680000
C 8.15030000 16.02630000 22.41320000
C 1.24240000 6.17620000 17.89030000
H 1.08310000 6.56170000 18.77700000
H 1.40320000 5.21320000 17.97690000
H 0.45870000 6.32700000 17.32230000
C 3.02250000 22.73570000 18.81840000
H 2.42950000 22.49080000 18.07770000
H 3.02690000 23.70990000 18.92490000
H 2.70250000 22.31810000 19.64510000
C -1.63830000 17.33200000 21.38160000
C 4.39640000 13.45530000 11.18860000
H 4.88540000 14.12760000 10.72930000
C 5.35350000 22.71060000 19.65410000
H 5.04730000 22.31780000 20.49780000
H 5.32430000 23.68810000 19.71900000
H 6.27360000 22.42310000 19.47500000
C 9.35920000 15.19090000 21.96590000
H 9.05670000 14.30230000 21.68670000

H 9.98910000 15.10340000 22.71140000
H 9.80310000 15.63670000 21.21400000
C 2.69550000 6.22380000 15.88260000
H 1.92070000 6.40320000 15.30960000
H 2.81650000 5.25570000 15.97430000
H 3.49750000 6.61680000 15.47970000
C 2.98350000 11.46110000 12.49910000
H 2.51050000 10.77540000 12.95580000
C 7.48500000 15.36000000 23.62800000
H 6.71010000 15.89250000 23.90790000
H 8.12800000 15.30820000 24.36530000
H 7.19050000 14.45810000 23.38580000
C 4.12390000 12.27450000 10.53910000
H 4.41730000 12.14300000 9.64500000
C -3.09310000 12.92420000 20.84280000
H -2.46720000 12.37460000 21.35910000
H -3.95760000 12.95480000 21.30270000
H -2.73590000 13.83320000 20.75800000
C 3.67110000 6.55740000 18.16210000
H 4.48470000 6.89740000 17.73420000
H 3.75480000 5.59110000 18.30160000
H 3.54490000 7.00300000 19.02520000
C 2.14220000 9.84690000 23.41410000
H 3.11040000 9.76880000 23.28020000
H 1.82050000 9.07410000 23.92400000
H 1.94630000 10.66940000 23.90860000
C 3.41770000 11.27250000 11.19960000
H 3.23240000 10.45380000 10.75460000
C 8.67370000 17.40960000 22.84830000
H 9.04940000 17.87570000 22.07220000
H 9.36980000 17.29630000 23.52910000
H 7.93590000 17.93620000 23.22000000
C -1.27440000 18.72770000 21.89340000
H -0.63920000 18.64780000 22.63570000
H -2.08380000 19.18410000 22.20370000
H -0.86510000 19.24490000 21.16820000
B 4.29380000 14.94610000 13.37780000
C -2.21670000 16.54530000 22.56230000
H -2.49200000 15.65560000 22.25790000
H -2.99360000 17.02210000 22.92280000
H -1.53510000 16.45630000 23.26000000

C 4.94480000 22.89110000 17.21590000
H 5.85460000 22.57930000 17.02490000
H 4.94770000 23.86620000 17.31210000
H 4.35220000 22.63780000 16.47690000
C -3.77430000 10.87540000 19.58930000
H -3.87100000 10.47290000 18.70150000
H -4.64310000 10.87520000 20.04430000
H -3.13000000 10.35880000 20.11540000
C -0.06750000 9.94870000 22.23860000
H -0.29610000 10.71000000 22.81290000
H -0.37890000 9.11940000 22.65710000
H -0.49870000 10.05720000 21.36590000
C 1.76190000 8.58340000 21.30380000
H 1.35570000 8.61160000 20.41170000
H 1.40160000 7.82130000 21.80250000
H 2.73370000 8.48660000 21.21580000
C -4.33500000 13.12380000 18.68640000
H -4.05130000 14.06010000 18.63140000
H -5.19020000 13.06880000 19.16160000
H -4.43860000 12.75970000 17.78290000
C -2.71360000 17.45360000 20.29150000
H -2.37870000 18.01830000 19.56420000
H -3.52100000 17.85920000 20.67360000
H -2.92870000 16.56410000 19.94360000

Cartesian coordinates of optimized 5TBO:
O 5.02050000 16.82850000 15.45670000
O 2.75910000 12.73190000 14.41820000
N 1.82220000 12.51320000 16.91060000
N 2.66120000 14.47930000 18.88750000
N 4.42590000 16.54670000 18.10100000
C 4.25910000 15.74960000 15.79500000
C 2.60190000 13.29610000 19.65090000
C 0.06260000 11.37330000 17.82460000
C 0.55450000 12.64720000 17.47580000
C 3.89010000 14.80420000 14.83260000
C 3.13750000 13.71850000 15.28000000
C 2.69640000 13.58280000 16.58710000
C 3.09140000 14.55310000 17.53690000
C 3.92030000 15.62550000 17.14430000
C 1.06940000 10.41210000 17.41790000

C 5.75720000 17.41350000 19.76030000
C 5.30950000 16.22450000 19.14490000
C 3.28900000 12.10350000 19.48100000
H 3.98700000 12.02430000 18.84070000
C 6.66340000 17.36070000 20.80930000
H 6.96010000 18.16700000 21.21430000
C 4.32490000 17.94930000 18.03100000
C -1.17520000 11.25560000 18.43380000
H -1.50470000 10.39620000 18.66970000
C 1.72590000 15.38890000 19.45800000
C 2.13690000 11.13970000 16.87050000
C -1.94210000 12.38330000 18.70310000
C 5.12900000 18.50890000 19.03460000
C 1.66530000 13.44580000 20.67390000
C 1.30970000 16.63140000 19.00400000
H 1.73310000 17.05460000 18.26620000
C -0.20730000 13.78590000 17.68080000
H 0.10560000 14.63940000 17.40460000
C 2.92290000 11.03060000 20.28040000
H 3.36920000 10.20120000 20.15720000
C 5.18440000 19.89400000 19.20380000
H 5.72510000 20.26980000 19.88820000
C 1.11320000 14.78010000 20.56670000
C 6.65050000 14.97400000 20.64830000
H 6.95680000 14.13270000 20.96610000
C 5.30570000 17.13120000 14.13360000
C 5.75050000 14.99090000 19.59930000
H 5.44440000 14.18380000 19.20260000
C 1.15660000 9.02450000 17.53320000
H 0.42750000 8.53340000 17.89340000
C -1.43350000 13.64470000 18.29750000
H -1.95740000 14.42110000 18.45620000
C 7.13900000 16.14570000 21.27300000
C 4.99660000 16.27950000 13.05430000
C 3.28810000 10.49960000 16.44250000
H 4.01240000 10.98790000 16.06780000
C 3.24960000 12.66450000 13.12020000
C 1.92570000 11.11140000 21.26050000
C 3.57750000 18.75640000 17.18440000
H 3.03590000 18.38110000 16.50020000
C 3.64840000 20.11620000 17.36890000

H 3.13660000 20.67650000 16.79710000
C 4.44230000 20.72130000 18.36590000
C 0.06270000 15.41740000 21.22060000
H -0.33670000 15.00620000 21.97830000
C 2.29960000 8.36100000 17.12560000
C 0.25180000 17.23200000 19.67020000
H -0.04720000 18.08150000 19.36720000
C 1.34410000 12.36010000 21.48740000
H 0.72600000 12.47060000 22.20040000
C -3.27690000 12.31420000 19.44480000
C 5.97220000 17.98800000 11.60330000
C 3.97340000 13.70690000 12.51460000
C -0.40900000 16.64460000 20.77970000
C 3.34830000 9.12410000 16.58050000
H 4.13360000 8.67390000 16.29330000
C 2.46380000 6.84030000 17.25940000
C 5.34830000 16.77340000 11.77460000
H 5.14620000 16.24860000 11.00880000
C 5.93520000 18.36270000 13.98250000
H 6.13460000 18.90200000 14.73880000
C 1.44240000 9.88870000 22.05510000
C 6.26420000 18.78330000 12.71320000
H 6.69540000 19.62140000 12.59150000
C 4.44550000 22.24810000 18.51680000
C 8.15030000 16.02630000 22.41320000
C 1.24240000 6.17620000 17.89030000
H 1.08310000 6.56170000 18.77700000
H 1.40320000 5.21320000 17.97690000
H 0.45870000 6.32700000 17.32230000
C 3.02250000 22.73570000 18.81840000
H 2.42950000 22.49080000 18.07770000
H 3.02690000 23.70990000 18.92490000
H 2.70250000 22.31810000 19.64510000
C -1.63830000 17.33200000 21.38160000
C 4.39640000 13.45530000 11.18860000
H 4.88540000 14.12760000 10.72930000
C 5.35350000 22.71060000 19.65410000
H 5.04730000 22.31780000 20.49780000
H 5.32430000 23.68810000 19.71900000
H 6.27360000 22.42310000 19.47500000
C 9.35920000 15.19090000 21.96590000

H 9.05670000 14.30230000 21.68670000
H 9.98910000 15.10340000 22.71140000
H 9.80310000 15.63670000 21.21400000
C 2.69550000 6.22380000 15.88260000
H 1.92070000 6.40320000 15.30960000
H 2.81650000 5.25570000 15.97430000
H 3.49750000 6.61680000 15.47970000
C 2.98350000 11.46110000 12.49910000
H 2.51050000 10.77540000 12.95580000
C 7.48500000 15.36000000 23.62800000
H 6.71010000 15.89250000 23.90790000
H 8.12800000 15.30820000 24.36530000
H 7.19050000 14.45810000 23.38580000
C 4.12390000 12.27450000 10.53910000
C -3.09310000 12.92420000 20.84280000
H -2.46720000 12.37460000 21.35910000
H -3.95760000 12.95480000 21.30270000
H -2.73590000 13.83320000 20.75800000
C 3.67110000 6.55740000 18.16210000
H 4.48470000 6.89740000 17.73420000
H 3.75480000 5.59110000 18.30160000
H 3.54490000 7.00300000 19.02520000
C 2.14220000 9.84690000 23.41410000
H 3.11040000 9.76880000 23.28020000
H 1.82050000 9.07410000 23.92400000
H 1.94630000 10.66940000 23.90860000
C 3.41770000 11.27250000 11.19960000
H 3.23240000 10.45380000 10.75460000
C 8.67370000 17.40960000 22.84830000
H 9.04940000 17.87570000 22.07220000
H 9.36980000 17.29630000 23.52910000
H 7.93590000 17.93620000 23.22000000
C -1.27440000 18.72770000 21.89340000
H -0.63920000 18.64780000 22.63570000
H -2.08380000 19.18410000 22.20370000
H -0.86510000 19.24490000 21.16820000
B 4.29380000 14.94610000 13.37780000
C -2.21670000 16.54530000 22.56230000
H -2.49200000 15.65560000 22.25790000
H -2.99360000 17.02210000 22.92280000
H -1.53510000 16.45630000 23.26000000

C 4.94480000 22.89110000 17.21590000
H 5.85460000 22.57930000 17.02490000
H 4.94770000 23.86620000 17.31210000
H 4.35220000 22.63780000 16.47690000
C -3.77430000 10.87540000 19.58930000
H -3.87100000 10.47290000 18.70150000
H -4.64310000 10.87520000 20.04430000
H -3.13000000 10.35880000 20.11540000
C -0.06750000 9.94870000 22.23860000
H -0.29610000 10.71000000 22.81290000
H -0.37890000 9.11940000 22.65710000
H -0.49870000 10.05720000 21.36590000
C 1.76190000 8.58340000 21.30380000
H 1.35570000 8.61160000 20.41170000
H 1.40160000 7.82130000 21.80250000
H 2.73370000 8.48660000 21.21580000
C -4.33500000 13.12380000 18.68640000
H -4.05130000 14.06010000 18.63140000
H -5.19020000 13.06880000 19.16160000
H -4.43860000 12.75970000 17.78290000
C -2.71360000 17.45360000 20.29150000
H -2.37870000 18.01830000 19.56420000
H -3.52100000 17.85920000 20.67360000
H -2.92870000 16.56410000 19.94360000
C 4.59944028 12.06136589 9.08995015
C 3.40887645 12.05832929 8.16580207
C 5.38014092 10.77525571 9.00127838
H 2.84783613 13.02010868 8.24969243
H 3.73443692 11.93106544 7.10501338
H 2.71471776 11.22136513 8.42088007
H 6.25553231 10.80199364 9.69391502
H 4.73735863 9.90484566 9.27825859
H 5.75699827 10.61452498 7.96221718
C 6.34463500 18.47020361 10.18895498
C 7.84340988 18.57933740 10.07378205
C 5.66460479 19.78624592 9.91141737
H 8.32332235 17.59316112 10.28345748
H 8.13657781 18.90300607 9.04579882
H 8.24074014 19.32717350 10.80199900
H 4.55687767 19.67961856 10.00275063
H 6.00508033 20.56552067 10.63550854

H 5.90107804 20.14140056 8.87921120

C 5.82988971 17.40768945 9.20016736

H 6.32469674 16.47748027 9.38665297

H 4.77499386 17.28251935 9.32833594

H 6.03233100 17.72482872 8.19849865

C 5.51907194 13.24553966 8.73836785

H 4.94956607 14.15138856 8.73745969

H 6.30180120 13.31706853 9.46439714

H 5.94481314 13.09093159 7.76896557

7.NMR spectroscopy

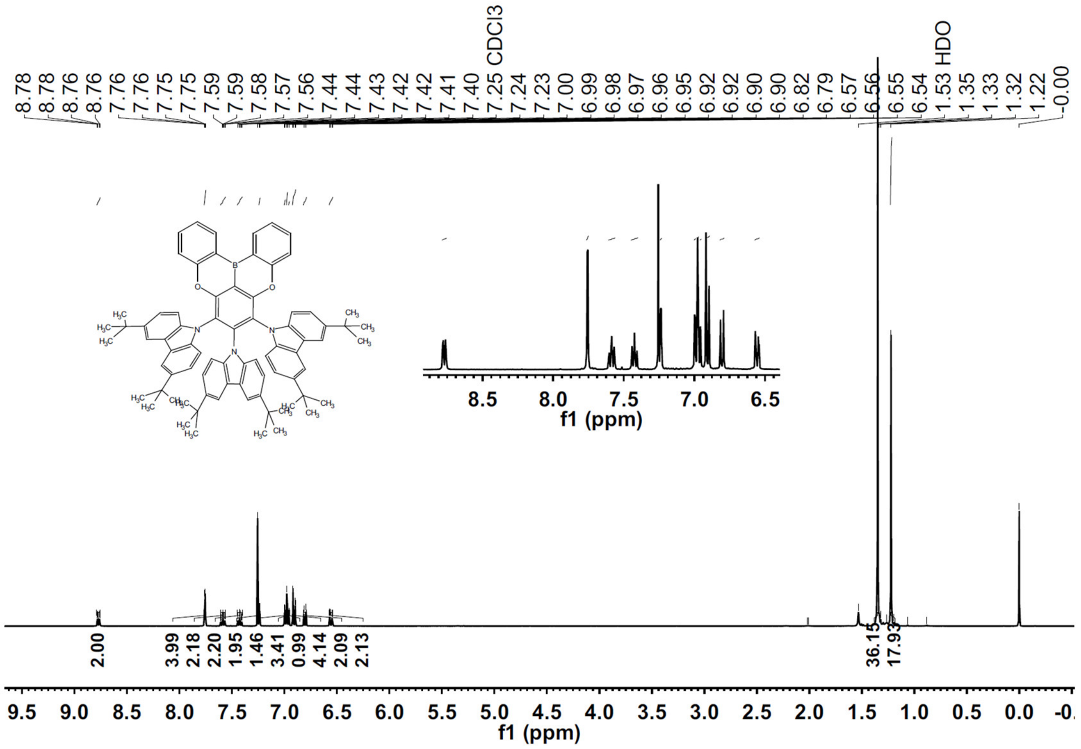

Figure S6. ^{1}H NMR spectrum of 3TBO in $CDCl_3$.

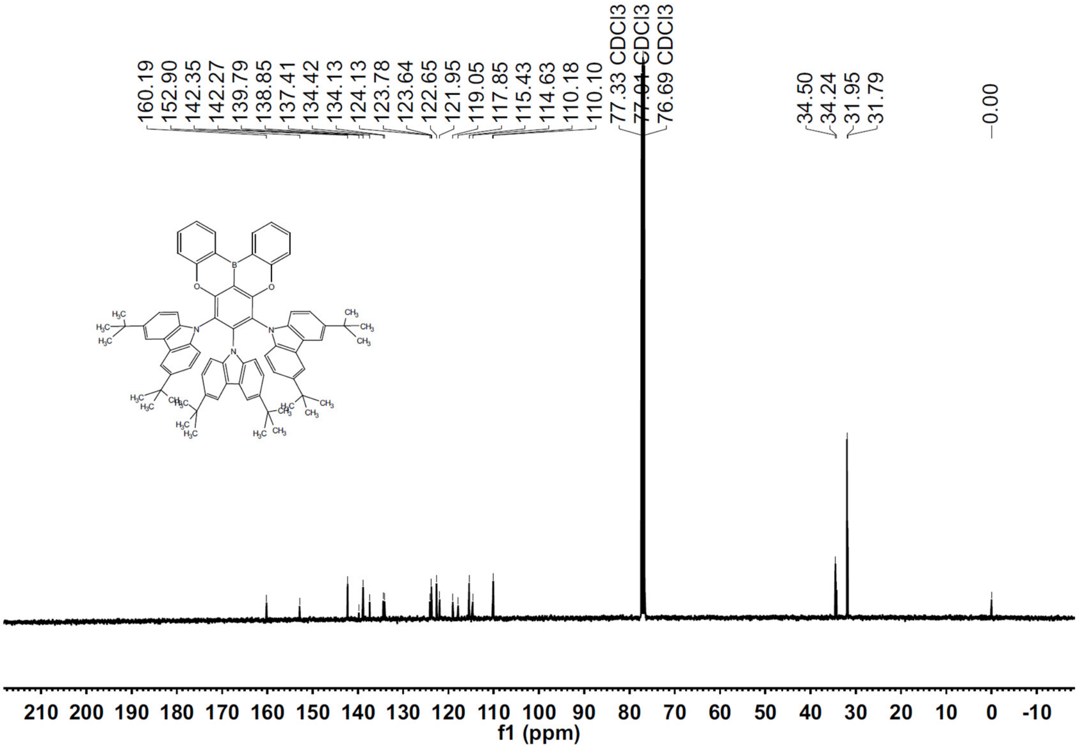

Figure S7. ^{13}C NMR spectrum of 3TBO in $CDCl_3$.

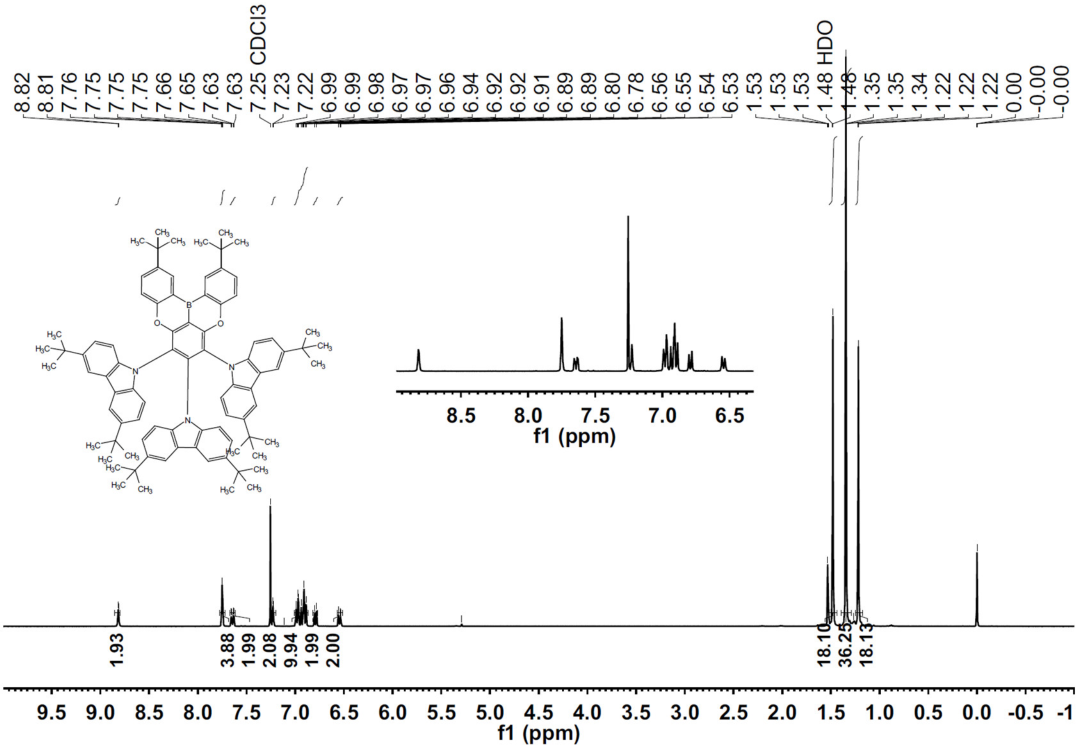

Figure S8. ^{1}H NMR spectrum of 5TBO in $CDCl_3$.

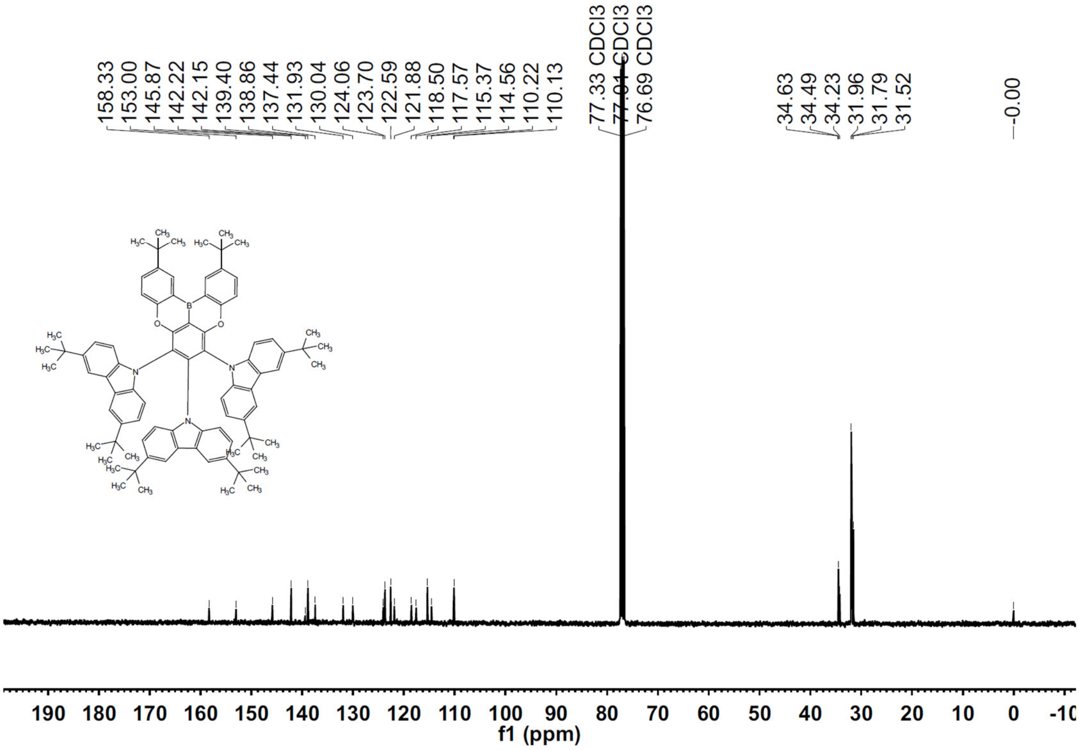

Figure S9. ^{13}C NMR spectrum of 5TBO in $CDCl_3$.
